# In Vitro and In Silico Studies of the Molecular Interactions of Epigallocatechin-3-*O*-gallate (EGCG) with Proteins That Explain the Health Benefits of Green Tea

**DOI:** 10.3390/molecules23061295

**Published:** 2018-05-28

**Authors:** Koichi Saeki, Sumio Hayakawa, Shogo Nakano, Sohei Ito, Yumiko Oishi, Yasuo Suzuki, Mamoru Isemura

**Affiliations:** 1 Section of Cell Engineering, ID Pharma Co. Ltd., Tsukuba, Ibaraki 300-2611, Japan; ks.saeki@gmail.com; 2 Department of Biochemistry and Molecular Biology, Graduate School of Medicine, Nippon Medical School, Tokyo 113-8602, Japan; hayakawa_sci@icloud.com (S.H.); y-oishi@nms.ac.jp (Y.O.); 3 Graduate Division of Nutritional and Environmental Sciences, University of Shizuoka, Shizuoka 422-8526, Japan; snakano@u-shizuoka-ken.ac.jp (S.N.); itosohei@u-shizuoka-ken.ac.jp (S.I.); 4 Department of Nutrition Management, Faculty of Health Science, Hyogo University, Kakogawa 675-0195, Japan; suz-cbio@hyogo-dai.ac.jp

**Keywords:** catechin, EGCG, affinity chromatography, binding interaction, health benefits, protein, green tea, molecular docking, cancer, epidemiology

## Abstract

Green tea has been shown to have beneficial effects on many diseases such as cancer, obesity, inflammatory diseases, and neurodegenerative disorders. The major green tea component, epigallocatechin-3-*O*-gallate (EGCG), has been demonstrated to contribute to these effects through its anti-oxidative and pro-oxidative properties. Furthermore, several lines of evidence have indicated that the binding affinity of EGCG to specific proteins may explain its mechanism of action. This review article aims to reveal how EGCG-protein interactions can explain the mechanism by which green tea/EGCG can exhibit health beneficial effects. We conducted a literature search, using mainly the PubMed database. The results showed that several methods such as dot assays, affinity gel chromatography, surface plasmon resonance, computational docking analyses, and X-ray crystallography have been used for this purpose. These studies have provided evidence to show how EGCG can fit or occupy the position in or near functional sites and induce a conformational change, including a quaternary conformational change in some cases. Active site blocking, steric hindrance by binding of EGCG near an active site or induced conformational change appeared to cause inhibition of enzymatic activity and other biological activities of proteins, which are related to EGCG’s biological oligomer and formation of their toxic aggregates, leading to the prevention of neurodegenerative diseases and amyloidosis. In conclusion, these studies have provided useful information on the action of green tea/catechins and would lead to future studies that will provide further evidence for rational EGCG therapy and use EGCG as a lead compound for drug design.

## 1. Introduction

**Figure 1:**
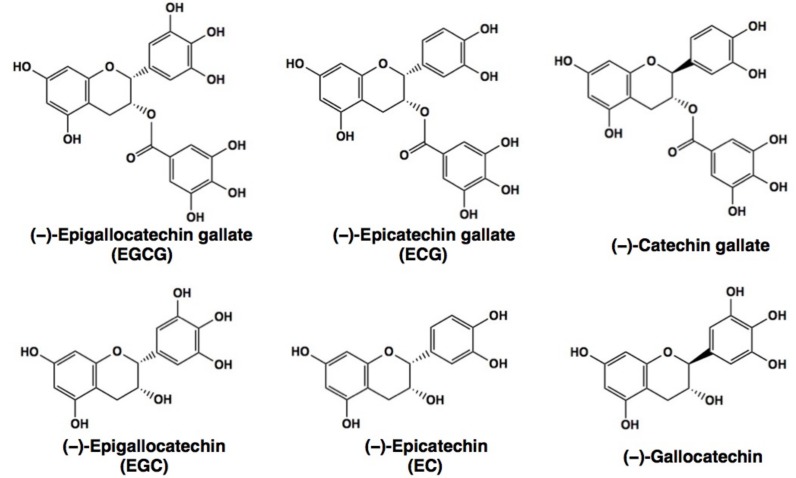
Chemical structures of major green tea catechins, EGCG and related compounds.

Green tea has been shown to have beneficial effects on many diseases such as cancer, metabolic syndrome (MetS), inflammatory diseases, and neurodegenerative disorders [1,2,3,4,5,6,7]. The green tea component, (−)-epigallocatechin-3-*O*-gallate (EGCG, Figure 1), is believed to be a major contributor to these effects. Several human observational and intervention studies have demonstrated that consumption of green tea/catechins has favorable effects on a variety of diseases. For example, daily treatment of volunteers with high-grade prostate intraepithelial neoplasia with total of 600 mg green tea catechins (GTCs)/day for one year resulted in a 90% reduction of the prostate cancer risk as compared to the placebo groups [8]. It should be noted that a standardized green tea polyphenol preparation has been approved by the U.S. Food and Drug Administration as a medication to treat genital warts [9].

In addition, an intervention study demonstrated that decreases in the disease markers’ values including those related to MetS such as body weight, body mass index, waist circumference, visceral fat area, and subcutaneous fat area, of the subjects who consumed green tea containing 583 mg of catechins were greater than those of the group which consumed 96 mg of catechins [10]. Scholey et al. found that EGCG administration induced a significant overall increase in α, β and θ activity, and increased self-rated calmness and reduced self-rated stress [11]. These findings encourage further studies on green tea/catechins; clarification of the action mechanisms including those associated with catechins–protein interactions will provide useful information which contributes to public health and the development of new drugs.

EGCG is a well-known anti-oxidant, and many peer-reviewed, scientific journal publications have demonstrated that the health effects of green tea are attributable to the anti-oxidative properties of green tea catechins (GTCs, Figure 1) including EGCG [1,2,3,4,5,6,7]. For example, carcinogenesis is closely associated with reactive oxygen species (ROS) which cause DNA damage and activate nuclear factor-κB (NFκB) which modulates the expression of cancer-associated cytokines such as the tumor necrosis factor (TNF)α and enzymes such as cyclooxygenase (COX) and matrix metalloproteinases (MMPs) [1]. The ROS-scavenging action of GTCs would down-regulate NFκB leading to anti-carcinogenic, anti-invasive, and anti-metastatic actions. Down-regulation of TNFα through suppression of NFκB would lead to anti-cancer effects as demonstrated by Fujiki et al. who showed that EGCG and GTCs inhibited the growth of human lung cancer PC-9 cells [12]. EGCG’s inhibition of invasion and metastasis of cancer cells may be explained by its suppressive effect on the activities and the gene expression of MMPs which degrade collagens in the basement membrane [2,13]. Anti-apoptotic protein B-cell lymphoma 2 (Bcl2) is suppressed by EGCG through down-regulation of NFκB, which can explain the apoptosis-inducing property of EGCG in its anti-cancer effect [14]. Cao et al. found that the high-fat-diet (HFD)-induced increases in inflammatory TNFα levels and infiltrating CD68^+^ macrophage counts in the rat islets were attenuated by supplementation of EGCG, suggesting that EGCG’s anti-diabetic effect may be by suppressing inflammation, by modulations, including suppression of NFκB activity [15].

Meanwhile, EGCG can act as a pro-oxidant. Several lines of evidence have shown that EGCG can stimulate ROS generation leading to the activation of AMP-activated protein kinase (AMPK) by phosphorylation. Phosphorylated AMPK generated by EGCG’s action can modulate some proteins involved in adipogenesis, lipogenesis, and lipolysis. This is the so-called ‘AMPK hypothesis’ proposed by Yang et al. [4]. For example, EGCG can exert an anti-obesity effect through the activation of AMPK, leading to the down-regulation of sterol-response element binding proteins (SREBPs) which are the transcriptional factors to up-regulate the gene expression of two lipogenic enzymes: fatty acid synthase (FASN) and 3-hydroxy-3-methyglutaryl-CoA reductase (HMGR). These proteins are known as targets for cancer therapy [16,17]. EGCG inhibits the expression of gluconeogenic enzymes glucose-6-phosphatase and phosphoenolpyruvate carboxykinase through AMPK activation leading to its anti-diabetic effect [1]. The weak effects of (−)-epicatechin (EC) and (−)-epigallocatechin (EGC) can be explained by the absence of a gallate residue which plays an important role in this binding. Furthermore, the cancer preventive ability of EGCG may be due to cellular increase in ROS through EGCG’s catalase inhibition [18].

Moreover, several lines of evidence have demonstrated that the binding affinity of EGCG to proteins is involved in its action mechanism [3,4,5,6]. The present review aims to update information from such studies on EGCG-protein interaction and discuss the molecular mechanism by which green tea may exert its health-promoting actions.

## 2. Affinity Gel Chromatography (AGC) and Other Conventional Methods

### 2.1. Studies on the Interaction between Catechins and Blood Proteins

In 1989, Arora et al. examined the interaction of native and modified bovine serum albumin (BSA) with catechin using equilibrium dialysis, pH-metric, viscosity and spectrophotometric methods. The order of reactivity of catechin binding to proteins was found to be: esterified BSA > BSA > formylated BSA > acetylated BSA with log K values of 3.778, 3.879, 3.748 and 3.813 and free energy changes equal to −5.11, −5.16, −5.07 and −5.15 kcal/mole, respectively [19]. Later, using a quartz crystal microbalance (QCM), Minoda et al. showed that EC, EGC, (−)-epicatechin gallate (ECG), and EGCG bound to human serum albumin with Ka values of 2.14 × 10^3^ M^−1^, 2.84 × 10^3^ M^−1^, 1.38 × 10^5^ M^−1^, and 2.53 × 10^5^ M^−1^, respectively. These values indicate that the affinity of the gallated catechins (ECG and EGCG) toward human serum albumin is about 100 times stronger than the affinities of un-gallated catechins (EC and EGC) [20].

In 1996, our research group demonstrated that among human plasma proteins, fibronectin (Table 1), fibrinogen, and histidine-rich glycoprotein, were specifically bound by EGCG immobilized on agarose gel [21]. These proteins appeared to have higher affinity to immobilized EGCG than plasma albumin, since in this AGC albumin was absent in the bound fractions which were obtained after washing with buffer containing 100 mM NaCl. Dot binding assays confirmed the interaction of EGCG with these proteins and showed that only gallated catechins were bound by these proteins [21]. EGCG bound to the C-terminal heparin-binding domain among the several fragments generated by thermolysin protein digestion, illustrating the specificity of the binding (Table 1) [22]. Thereafter, the EGCG-protein interaction has often been demonstrated by using EGCG-conjugated AGC.

EGCG-conjugated agarose gel has also been utilized as a pull-down agent to show the binding interaction between EGCG and proteins (Table 1). These proteins include heat shock protein 90 (HSP90) [23,24], glucose-regulated protein 78 kDa (GRP78) [25], insulin-like growth factor 1 receptor (IGF1R) [26], Fyn [27], ζ chain-associated 70-kDa protein (ZAP70) [28], Ras-GTPase-activating protein Src homology (SH3) domain-binding protein 1 (G3BP1) [29], peptidyl-prolyl cis-trans isomerase (Pin1) [30], and TNF receptor-associated factor 6 (TRAF6) [31].

### 2.2. Interaction between Catechins and Cancer-Related Protein

The result of AGC using the EGCG-agarose column provided convincing evidence that MMPs derived from cancer cells were bound by, and eluted from the column [32]. These MMPs were identified as MMP2 and MMP9 (Table 1) which have roles in the invasion and metastasis of cancer cells. Our research group has similarly demonstrated the binding interaction between EGCG and cell surface Fas protein [34], laminin, fibrinogen subunits, and integrin β1 [35,36,37]. These proteins are related to cancer, and EGCG binding would cause impairments in cancer development, which may be responsible for EGCG’s beneficial effects on cancer. The EGCG-binding interaction was also demonstrated for platelet-derived growth factor [38].

**Figure 2:**
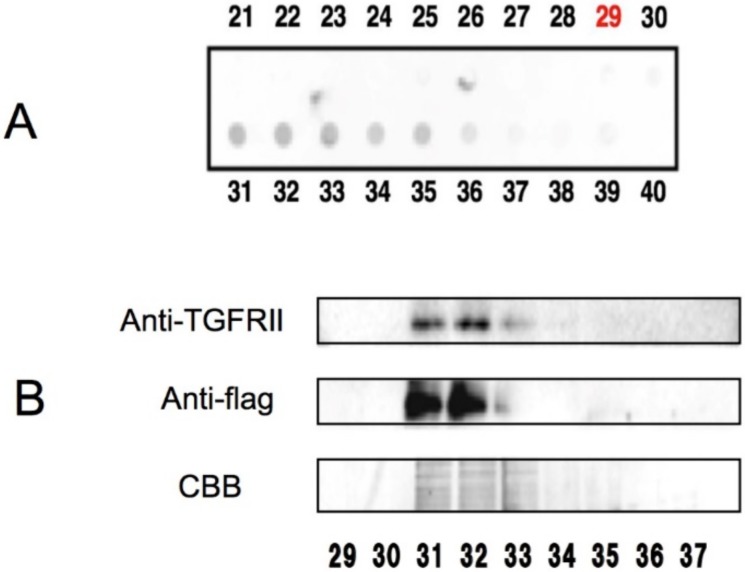
AGC showing the binding between EGCG and TGFβRII [39]. (**A**) Each of the fractions (fraction numbers 29–40) bound to and eluted from an EGCG-agarose column was spotted onto a membrane and stained with Coomassie brilliant blue (CBB) to detect proteins. (**B**) The EGCG-bound fractions (29–37) in A were examined by SDS-PAGE and immunoblotting with anti-TGFβR antibodies. Use kindly permitted by the publisher of *World Journal of Experimental Medicine*, Baishideng Publishing Group Inc. doi:10.5493/wjem.v3.i4.100.

More recently, Tabuchi et al. used AGC to study the interaction of EGCG and transforming growth factor (TGF)β type II receptor (TGFRII) [39]. When the cell lysates from the TGFRII-expressing COS-7 cells were examined by EGCG-AGC, TGFRII was found in the bound fractions as shown by western blotting using anti-TGFRII antibodies (Figure 2). Since EGCG was found to inhibit the expression of α-smooth muscle cell actin through the TGFβ-Smad2/3 pathway, this finding suggests an anti-fibrotic role for EGCG. The observation that TGFβ can enhance tumorigenicity [40] and inhibitors of TGFβ-signaling elicit anti-tumor effect in patients [41] suggests that EGCG binding to TGFβ might contribute to the green tea’s anti-tumor action.

In 2005, Ermacova et al. conducted EGCG-AGC of lysates from JB6 Cl41 cells and identified vimentin as one of the bound proteins (Table 1). EGCG inhibited phosphorylation of vimentin at Ser50 and Ser55 and inhibited cell proliferation. Because vimentin is important for maintaining cellular functions, the inhibitory effect of EGCG on vimentin may be contributing to the anti-cancer action of green tea [33].

The aryl hydrocarbon receptor (AhR) is a ligand-activated transcription factor known to mediate the toxic effects of numerous environmental contaminants such as the polycyclic aromatic hydrocarbons. EGCG is capable of antagonizing AhR-mediated gene transcription, leading to chemoprevention. Competitive binding assays suggest that EGCG does not bind at the AhR ligand binding site. Pull-down experiments with EGCG-agarose suggested a mechanism of action involving direct binding of EGCG to the AhR chaperone protein, HSP90. The amino-terminal fragment containing the geldanamycin binding site (Cys507) did not bind to EGCG-agarose. Three C-terminal truncation mutants of HSP90-bound EGCG and analysis of the smallest mutant polypeptide containing amino acid residues 538–728 showed that the binding of EGCG occurs within this region of the protein, suggesting an interaction with the C-terminal ATP binding site. The binding induces an AhR conformation capable of nuclear localization but incapable of binding DNA. These data suggest that EGCG directly binds HSP90 and exerts its chemopreventive effects through an interaction with chaperone HSP90 protein [23].

Moses et al. found that in mice treated with tumorigenic or metastatic cells, EGCG supplementation caused smaller sized tumors than in untreated mice, and that EGCG prevented malignant transformation in the animal model of prostate cancer. Binding assays with EGCG-agarose, a C-terminal HSP90 antibody, and HSP90 mutants showed that EGCG bound more HSP90 from metastatic cells in contrast to non-tumorigenic cells, and the binding occurred through the HSP90 C-terminus. Also, EGCG-agarose bound to wild-type HSP90α, HSP90α with E47A mutation, and HSP90α with D93A mutation, suggesting that the EGCG binding is independent of either ATP-dependent alterations in N-domain conformation or by N-domain binding-pocket nucleotide occupancy. Consistent with HSP90 inhibitory activity, EGCG, novobiocin, and 17-allylamino-17-demethoxy-geldanamycin induced changes in HSP90-client proteins in non-tumorigenic cells and larger differences in metastatic cells. These data suggest that EGCG may be efficacious for the treatment of prostate cancer because it preferentially targets cancer cells and inhibits a malignant phenotype supportive molecular chaperone [24].

He et al. found that EGCG inhibited epidermal growth factor (EGF)-induced transformation of JB6 Cl41 cells and inhibited EGF-induced Fyn kinase activity [25]. The EGCG pull-down assay indicated that Fyn bound to EGCG-agarose gel, and that EGCG bound to the glutathione-S-transferase (GST)-Fyn (SH2 domain) fusion protein but not GST-Fyn (SH3 domain) fusion protein. The SH2 domain of Fyn bound to EGCG with a Kd value of 0.367 ± 0.122 μM. Based on these and other findings, the authors proposed that EGCG inhibits the EGF-induced transformation through inhibition of EGF-induced Fyn phosphorylation, which causes p38 MAPK activation and subsequent activation of STAT1, ATF-2, and AP-1 [27]. The proposal may explain EGCG’s contribution to the anti-cancer effect of green tea.

Ras-GTPase-activating protein (GAP) modulates the downstream signal transduction of Ras, one of the critical oncoproteins in carcinogenesis. When lung cancer cell lysates were precipitated with EGCG-agarose gel beads, western blot analysis demonstrated G3BP1 binding affinity to EGCG. The Kd of G3BP1 for EGCG was 0.4 μM. Pull-down experiments showed EGCG’s binding to mutants such as G3BP1m (1–415), G3BP1m2 (1–340), but not to G3BP1m3 (1–225) or G3BP1m6 (1–225, 341–415). These data indicate that EGCG interacts with the Ras-GAP–binding region and the glycine-rich domain of G3BP1. EGCG suppressed the anchorage-independent growth of G3BP1-overexpressing H1299 and CL13 lung cancer cells. EGCG was much less effective in suppressing the anchorage-independent growth of H460 lung cancer cells, which express much lower levels of G3BP1. EGCG interfered with the interaction of G3BP1 and the Ras-GAP, and further suppressed the activation of Ras. Additionally, EGCG effectively attenuated G3BP1 downstream signaling, including extracellular signal-regulated kinase and mitogen-activated protein kinase/extracellular signal-regulated kinase kinase, in wild-type H1299 and shMock H1299 cells. These results indicate that EGCG suppresses lung tumorigenesis through its binding with G3BP1 [29].

Urusova et al. performed a pull-down experiment to show that EGCG bound Pin1, and that EGCG suppressed tumor growth in a xenograft study using mice injected with Neu/Pin1 wild-type or knockout cells and tumor formation in nude mice injected with Pin1 expressing HCT116 colorectal cancer cells [30].

TNF receptor-associated factor 6 (TRAF6) is an E3 ubiquitin ligase that contains a RING (really interesting new gene) domain, induces K63-linked polyubiquitination, and plays a critical role in signaling transduction. TRAF6 is overexpressed in melanoma, and TRAF6-knockdown dramatically attenuates tumor cell growth and metastasis. When cell lysates from TRAF-expressing cells were examined, a pull-down experiment and western blotting analysis showed that TRAF6 bound to the EGCG-conjugated agarose beads but not to the agarose beads itself [31].

Tanaka et al. utilized boronate-bound agarose gel to pull-down EGCG binding proteins in AZ521 cells and identified the DEAD-box RNA helicase p68, which is overexpressed in tumor cells and plays an important role in cancer development and progression. EGCG decreased the concentration of p68 in these cells. These and other results suggested that EGCG inhibited gastric cancer AZ521 cell proliferation by preventing β-catenin oncogenic signaling through proteasomal degradation of p68, a new perspective on the molecular mechanism of the EGCG’s action [42].

### 2.3. Interaction between Catechins and Proteins Related to Cardiac Muscle Disease and Amyloid Disease

Tadano et al. investigated the direct actions of GTCs on cardiac muscle function to explore their uses as potential drugs for cardiac muscle disease using techniques including QCM and NMR. EGCG and ECG, but not their stereoisomers (−)-catechin-3-gallate and (−)-gallocatechin-3-gallate, decreased cardiac myofilament Ca^2+^ sensitivity probably through their interaction with cardiac troponin C. EGCG restored cardiac output in a mouse model of hypertrophic cardiomyopathy. EGCG and ECG may have the beneficial effect on the hypertrophic cardiomyopathy caused by increased Ca^2+^ sensitivity of cardiac myofilaments [43].

Kamihira-Ishijima et al. investigated the inhibition mechanism of GTCs on amyloid fibril formation of the islet amyloid polypeptide fragment (F22–27), which is of sufficient length for the formation of β-sheet-containing amyloid fibrils. QCM determined that the association constants of ECG and EGCG for binding to F22–27 immobilized on the gold plate were one order of magnitude higher than the affinity of the free F22–27 peptide. NMR confirmed the formation of an F22–27/ECG complex. These findings suggest that EGCG and ECG inhibit the early stage amyloid fibril formation to generate amyloid nuclei, leading to the prevention of amyloid diseases [44].

## 3. Surface Plasmon Resonance (SPR)

### 3.1. Interaction between Catechins and Proteins Related to Cancer, Inflammatory Disease, and Oral Health

In 2004, Tachibana et al. discovered the cell surface 67-kDa laminin receptor (67LR) as the receptor of EGCG by an SPR study [45]. The binding of EGCG to the protein occurs at physiologically available concentrations and has been shown to mediate many of EGCG’s beneficial activities, including anti-cancer, anti-diabetic, anti-atherosclerosis, anti-allergic, and anti-inflammatory activities. Myosin phosphatase target subunit, eukaryotic elongation factor 1A, protein phosphatase 2A, AKT/protein kinase B, endothelial nitric oxide synthase, soluble guanylate cyclase, protein kinase C, acid sphingomyelinase, sphingosine kinase 1, and cGMP have been identified as EGCG-sensing associated molecules [46].

Later, Fujimura et al. found that EGCG cell surface binding and cancer cell growth were both inhibited by a peptide containing the 10-amino acid residues, Ile-Pro-Cys-Asn-Asn-Lys-Gly-Ala-His-Ser, corresponding to residues 161–170 in 67LR and that EGCG formed a complex with the 161–170 peptide [47]. These authors also showed the critical role of the 10-amino acid length and two basic amino acids, Lys166 and His169, in EGCG’s activities and proposed this amino acid sequence to be the functional domain responsible for the anti-cancer activity of EGCG. It is interesting to note that EGCG inhibits the binding of laminin to 67LR, but the 67LR’s EGCG binding site (161–170) is separated by a short distance from the laminin-binding 173–178 site.

Furthermore, Tsukamoto et al. demonstrated that EGCG-induced apoptosis involved 67 LR-mediated clustering of lipid-rafts which are cholesterol- and sphingolipid-rich membrane domains [48]. Acid sphingomyelinase (aSMase) plays a critical role for the lipid-raft clustering and was shown to be activated by EGCG in a murine multiple myeloma xenograft model. EGCG induced aSMase translocation to the plasma membrane and protein kinase Cδ phosphorylation at Ser664, which was necessary for aSMase/ceramide signalling via 67LR [48]. These findings suggest that the lipid-raft clustering/67LR/aSMase can be a novel therapeutic target against multiple myeloma and possibly other types of cancer. More detailed information on 67LR is available in the comprehensive review articles by Tachibana [46,49] and Yang and Wang [6].

In a study into the effects of four GTCs on protein ERp57, known as protein disulfide isomerase isoform A3, the interaction of catechins with ERp57 was examined by fluorescence quenching and SPR techniques. EGCG and ECG had a higher affinity than non-gallated catechins. EGCG bound the site close to the protein’s thioredoxin-like redox-sensitive active sites. The effects of these catechins on ERp57 properties were also investigated, and moderate inhibition of the reductase activity of ERp57 and a strong inhibition of ERp57 DNA binding activity were observed. Considering the high affinity of gallated catechins for ERp57 and their capability to inhibit ERp57 binding to other macromolecular ligands, some effects of the catechin interaction with this protein on eukaryotic cells may be expected. This study provides information to better understand the molecular mechanisms underlying the biological activities of catechins and to design new polyphenol-based ERp57-specific inhibitors [50].

### 3.2. Interaction between Catechins and Proteins Related to Inflammatory Disease and Oral Health

CD4^+^ Th1 cells play critical roles in host defense against pathogens and the pathogenesis of many immune-mediated diseases. Qin et al. found that i.p. injection of EGCG efficiently decreased the recruitment of murine ovalbumin-specific Th1 cells and other inflammatory cells into the airways in a Th1 adoptive-transfer model of SCID mice. Experiments with chemokines, C-X-C motif chemokine ligand (CXCL)9, CXCL10 and CXCL11 showed that EGCG inhibited chemotaxis of primary human CD4^+^ cells and murine cells stably expressing C-X-C motif chemokine receptor 3, whereas EC and EGC exhibited marginal inhibitory activity. SPR studies showed that EGCG bound directly to these chemokines, whereas binding of EC or EGC was much weaker. These findings suggest that EGCG’s direct binding of pro-inflammatory chemokines and resultant inhibition of recruit of inflammatory cells is involved in its anti-inflammatory mechanism and warranted further development of EGCG as a potent therapeutic for inflammatory diseases [51].

Tea interacts with saliva upon entering the mouth so that GTCs may regulate salivary proteins’ functions. Matrix-assisted laser desorption/ionization time-of-flight mass spectrometry peptide mass fingerprinting showed that the major proteins precipitated by EGCG were α-amylase, S100, and cystatins. EGCG bound to α-amylase at dissociation constant of Kd = 2.74 μM as determined by SPR and inhibited the activity by non-competitive inhibition, suggesting that EGCG can inhibit caries formation by inhibiting the formation of fermentable carbohydrates. EGCG-salivary protein interactions might have a role in oral health [52].

## 4. Computational MDA

### 4.1. Interaction between Catechins and Cancer-Related Proteins

There are several studies in which EGCG-protein interactions as revealed by AGC and pull-down experiments using EGCG-gel were further investigated by MDA (Table 1). As described above, in our previous studies, AGC demonstrated the binding interaction between EGCG and MMPs (MMP2 and MMP9) [32]. Later, EGCG and ECG, but not EC and EGC, were observed to inhibit pro-/active MMP2 activities [53].

Chowdhury et al. confirmed that among the four GTCs, EGCG and ECG inhibit pro-/active MMP2 activities in pulmonary artery smooth muscle cell culture supernatant and a gallate group is important for a strong interaction of pro-/active MMP2 with EGCG and ECG [53]. Studies using gelatin zymography and MDA showed that EGCG and ECG were better inhibitors for proMMP2 as compared with MMP2 and showed a strong interaction with MT1-MMP (MMP14) which is involved in the conversion of proMMP2 to active MMP2. These findings are consistent with our previous observation that EGCG and ECG inhibited the MMP2/MMP9-mediated gelatin degradation and the invasion of highly metastatic human fibrosarcoma HT1080 cells through an endothelial cell monolayer [13].

**Figure 3:**
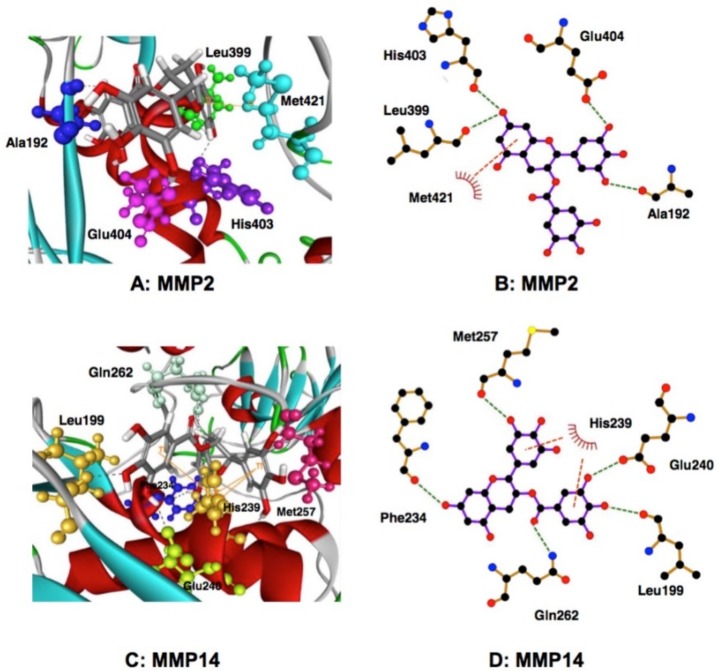
Interaction of EGCG with MMPs as examined by MDA [53]. Interactions in EGCG and MMP2 (**A**,**B**) and MMP14 (**C**,**D**) are shown. The green dotted lines represent the expected hydrogen bonds in a complex of EGCG and MMP2 (**B**) and MMP14 (**D**). The orange dotted lines show the expected π-interactions in the MMP2 (**B**) and MMP14 (**D**). Reproduction kindly permitted by Springer International Publishing AG, the publisher of *Molecular and Cellular Biochemstry*, doi:10.1007/s11010-016-2903-y.

MDA substantiated these findings, and EGCG and ECG were better inhibitors for proMMP2, compared with MMP2. The results showed that the 15 amino acid residues have interaction energy > 2 kcal/mol in the MMP2-EGCG complex, and Ala192, Leu399, His403, Glu404, Met421 are located close to the EGCG binding site (Figure 3) [53]. Pro423 appears to contribute to the complex formation with EC and EGC, but not with EGCG, and this interaction may deprive EC and EGC of their inhibitory activity against MMP2. Met421 may be involved in the π-interactions in the EGCG-MMP2 complex. In many cell types, proMMP2 is activated to form MMP2 by MMP14. MDA also showed a strong interaction of MMP14 with both EGCG and ECG, and the galloyl group responsible for the interaction. Glu240, Leu199, Gln262, Met257, Phe234, and His239 are involved in the interaction, the active site residue Glu240 displays high hydrogen bond interaction, and His239 may be involved in the interaction in the EGCG-MMP14 complex [53].

Similarly, Sarkar et al. demonstrated that among four GTCs, EGCG and ECG inhibited pro-/active MMP9 activities in the culture medium of cultured pulmonary artery smooth muscle cells. MDA showed a strong interaction between pro-/active MMP9 and both EGCG and ECG, and the galloyl group apears to contribute to this interaction [54]. These findings are important to better understand the mechanistic aspects of green tea in the prevention of diseases such as cancer, pulmonary hypertension, cardiovascular diseases, and neurological disorders [54].

By using a combination of NMR, fluorescence polarization assay, and MDA, Leone et al. found that GTCs and black tea theaflavins are potent inhibitors of the anti-apoptotic Bcl2-family proteins, Bcl2 and B-cell lymphoma-extra large (BclxL). ECG and (−)-catechin gallate showed the strongest inhibition with Ki values of 120 and 143 nM for BclxL, respectively. It may be noted that the corresponding value 490 nM for EGCG was much higher. On the contrary, GTCs lacking the gallate group showed no inhibition at 100 μM. Similar findings were obtained in Bcl2 as well. GCG, EGCG, CG, and ECG docked well in the BH3-binding pocket. When the binding region on the surface of BclxL is subdivided into three subpockets (P1, P2, and P3), the gallate moiety of GCG, EGCG, and CG fits mainly to the less lipophilic subpocket P1, with the exception of ECG in which the gallate was predicted to be located in the opposite subpocket P3. In contrast, compounds without the gallate group, such as GC, EGC, EC, and catechin did not dock well. Comparison of the docked structures of CG and catechin shows that catechin without the gallate ring occupies only one subpocket, namely P3, suggesting that the occupation of all three of these subpockets is required for tight binding and inhibition of the Bcl2 protein’s activity. GTCs with a galloyl group may promote apoptosis by binding to and inhibiting Bcl2-family proteins [55].

Glioblastoma (GBM) is a malignant form of brain tumor and has a high level of expression of GRP78, one of the HSP70 protein family members. GRP78 is the key chaperone protein involved in the unfolded protein response and has an ATPase domain, a substrate binding domain, and a linker region. EGCG is one of the ATP-competitive inhibitors and reduces its expression in GBM. Bhattacharjee et al. conducted MDA and identified unique amino acid residues in the ATPase domain of GRP78 which were different from those present in other HSP70 proteins. The data showed that amino acid residues in GRP78 such as Ile61, Glu293, Arg297, and Arg367 are involved in the intermolecular interactions with EGCG. Energetic contribution of individual inhibitor-interacting residues showed that energy values of Ile61 and Glu293 were among the strongest [56]. Drugs targeting GRP78 may be useful for potential therapy in GBM and other types of cancer [57].

Tyrosine kinase-type growth factor receptors regulate numerous biological processes, including cellular growth, proliferation, metabolism, survival, cell differentiation, and apoptosis, and can be the target for cancer therapy, given their overexpression in cancer cells. Singh et al. conducted MDA of several receptors, including IGF1R, VEGF1R, and VEGF2R against natural compounds. These compounds were docked with the X-ray crystal structure of selected proteins by employing Grid-based Ligand Docking with Energetics Maestro 9.6, and 20 compounds were selected by G-score along with 68 anti-cancer compounds. The results demonstrated that EGCG yielded a magnificent G-score with IGF1R and VEGF2R. EGCG reduced cell proliferation and ROS generation. EGCG also reduced cell migration, suggesting its anti-metastasis activity [58].

Tyrosine kinases regulate various biological processes and are drug targets for cancers. Hsu et al. presented an anchor-based classification for tyrosine kinases and discovered two type-C inhibitors, rosmarinic acid, and EGCG, which occupy two and one specific anchors, respectively, among 118,759 natural compounds screened. Rosmarinic acid and EGCG inhibited 3% and 14% of 64 species of kinases, respectively. MDA showed that EGCG forms hydrogen bonds with Ser720, Gln791, Met793, Asp800, and Glu804. These inhibitors maintained inhibitory activities for drug-resistant EGF receptor with mutations and decreased the invasion of breast cancer cells. These findings suggest that EGCG may be useful as a therapeutic agent against cancer types which have a high mutation rate [59].

ZAP70 of tyrosine kinase plays a critical role in T cell receptor-mediated signal transduction and the immune response. Its high level of expression in leukemia implies that ZAP70 may be a target for leukemia therapies. ZAP70 and EGCG displayed high binding affinity (Kd = 0.6207 μM), and EGCG dose-dependently induced caspase-mediated apoptosis in ZAP70-expressing leukemia cells, whereas ZAP70-deficient cells were resistant to EGCG treatment. MDA and site-directed mutagenesis experiments showed that EGCG forms a series of intermolecular hydrogen bonds and hydrophobic interactions within the ATP binding domain. The study indicates that EGCG inhibits ZAP70 activity, leading to the suppression of the CD3-mediated T cell-induced pathways in leukemia cells [28].

As described above, TRAF6 bound to the EGCG-conjugated agarose beads. MDA confirmed that EGCG can directly bind to TRAF6 and that EGCG interacts with TRAF6 at the residues of Gln54, Gly55, Asp57 ILe72, Cys73, and Lys96. Among these amino acids, mutation of Gln54, Asp57, ILe72 in TRAF6 could destroy EGCG binding to TRAF6. EGCG can form five hydrogen bonds with TRAF6: three involved in the side-chain atoms of three residues Gln54, Asp57, and Lys96, while the other two engaged with the backbone atoms of two residues, namely Gly55 and Ile72. These residues, located either within or nearby the RING domain of TRAF6, appear to play an important role in TRAF6’s strong binding with Ubc13. Thus, the MDA showed that EGCG can have the Ubc13-competitive inhibitory effect on TRAF6 protein. These results demonstrate that EGCG is a novel ubiquitin ligase inhibitor that could be used to target TRAF6 for chemotherapy or the prevention of melanoma [31].

Many other studies have also demonstrated the EGCG-protein interaction as shown below. The binding of GTCs may alter the conformation of enzyme proteins or block the interaction with the substrate, leading to changes in the stability and the enzyme activity of a variety of proteins. These include urokinase [60], trypsin, chymotrypsin, squalene epoxidase, ribonuclease, α-amylase, xanthin oxidase, and histidine/dopa decarboxylase [61].

Urokinase plays an important role in the invasion and metastasis of cancer cells, and its inhibition can decrease tumor size and cause remission of cancers. In 1997, MDA performed by Jankun et al. found that EGCG is well fitted into the enzyme cavity composed of the catalytic triad His57, Asp102, and Ser195 and two positively charged residues, namely Arg35 and Arg37 [60]. It should, however, be noted that the urokinase inhibition required mM levels of the EGCG concentration, which is far in excess of the physiologically available concentration.

Cui et al. studied the binding mode of GTCs to trypsin using methods, including MDA. The results indicated that GTCs with different structures bound to a conservative pocket S1 of trypsin, which is comprised of amino acid residues 189–195, 214–220 and 225–228. In the trypsin-catechin complexes, Asp189 by forming strong hydrogen bonding, and Gln192, Trp215, and Gly216 through hydrophobic interactions, all significantly contribute to the binding of GTCs. The number and the position of hydroxyl and aromatic groups, the structure of stereoisomers, and the orientation of GTCs in this pocket can affect the binding affinity. The binding affinity is in the order of EGCG > ECG > EC > EGC, and the 2R-3R stereoisomer of EGCG showed the strongest binding [61]. With all these GTCs, residues Ser190, Gln192, Ser195 and Val213-Ser214-Trp215 in common contribute to conserved hydrogen bond interactions or hydrophobic contacts. Phe41, Cys42, and Leu99 are involved in hydrophobic interactions and the backbone of Phe41 forms hydrogen bond interaction with ECG and EGCG. When GTCs occupy the catalytic pocket S1, the substrate binding to trypsin would be hindered, and its protein-degrading activity would be inhibited, leading to a nutritionally unfavorable result. However, the GTC’s effect on trypsin would lead to the prevention of the proliferation, invasion, and metastasis of cancer cells, which require degradation of the extracellular matrix proteins [61].

Proteasome inhibitors may be anti-cancer agents that induce tumor growth arrest and cell death. Smith et al. demonstrated that EGCG inhibited the chymotryptic activity of the 20S proteasome time-dependently and irreversibly, and implicated acylation of the β5-subunit’s catalytic N-terminal threonine (Thr1), consistent with the previous finding that EGCG and ECG were potent proteasome inhibitors. MDA revealed that EGCG bound the chymotrypsin site in an orientation and conformation that is suitable for a nucleophilic attack by Thr1. The distance from the electrophilic carbonyl carbon of EGCG to the hydroxyl group of Thr1 was 3.18 Å (Angstrom). The A ring of EGCG acts as a tyrosine mimic and binds to the hydrophobic S1 pocket of this subunit. During this binding process, the EGCG’s scissile bond may become strained, which could lower the activation energy for attack by the hydroxyl group of Thr1 [62].

To identify GTC-binding proteins related to human diseases, Zheng et al. used two reverse docking systems, one based on Autodock software and the other on Tarfisdock. These methods ranked potential target proteins by the binding energy score. To further analyze the validity of docking results, the binding mode of EGCG to leukotriene A4 hydrolase was examined in detail. The results indicated that interactions mediated by electrostatic and hydrogen bond play a key role in this binding. EGCG was found to bind to enzymes with a certain orientation and conformation that are suitable for nucleophilic attacks by several residues with negative ionizable groups, inside the enzyme’s activity cavity. This methodology would be useful to explore agents to target human diseases, including cancer [63].

EGCG has been shown to modulate many cancer-related pathways through epigenetic alterations, including DNA methylation. Hypermethylation of CpG islands in the promoter regions is an important mechanism to silence the expression of many genes. Using polydeoxyinosine-deoxycytosine as a substrate, Fang et al. demonstrated that EGCG inhibited DNA methyltransferase (DNMT) activity, showing competitive inhibition with a Ki of 6.89 μM [64]. MDA suggested that EGCG could form hydrogen bonds with Pro1223, Glu1265, Cys1225, Ser1229, and Arg1309 in the catalytic pocket of the enzyme. EGCG treatment of cancer KYSE 510 cells caused a reversal of hypermethylation of genes such as retinoic acid receptor-β and human mutL homolog 1 (hMLH1), being accompanied by the expression of mRNA and the re-expression of retinoic acid receptor-β and hMLH1 proteins. Perhaps, this paper is the first to show the inhibition of DNA methylation by a commonly consumed dietary constituent [64]. In a later study, Khan et al. showed that (1) EGCG caused a significant reduction in the enzymatic activity of DNMT and HDAC in HeLa cells; (2) the gallate moiety of EGCG occupies a binding pocket analogous to the pyrimidyl ring of cytosine to exert direct inhibition of the enzyme; and (3) the gallate moiety frequently binds in proximity to the active residue or the other key residues such as Arg832, Arg823, Ser778 and Asn652. These results suggest that EGCG may have a significant impact on the development of a novel epigenetic-based therapy [65].

Dihydrofolate reductase (DHFR) is the subject of intense investigation because it is the primary target enzyme for anti-folate drugs such as methotrexate. Fluorescence quenching experiments show that EGCG and ECG are potent inhibitors of DHFR with Kd = 0.9 and 1.8 μM, respectively, whereas EGC and EC showed no binding. When 3-*O*-(3,4,5-trimethoxybenzoyl)-EC, a synthetic derivative of ECG, which effectively binds to DHFR (Kd = 2.1 μM), was used, the derivative generated a stable quinone methide product in alkaline solution and strongly bound to the enzyme with Kd of 8.2 nM [66]. The result may explain the anti-cancer effect of green tea.

The phosphoinositide-3-kinase (PI3K) signaling pathway is activated in many human cancers and may be an important therapeutic target for the treatment of cancer. Van Aller et al. showed that EGCG is an ATP-competitive inhibitor of both PI3K and mammalian target of rapamycin with Ki of 380 and 320 nM, respectively. EGCG inhibited cell proliferation and AKT phosphorylation at Ser473 in MDA-MB-231 and A549 cells. MDA showed that EGCG binds to the PI3K kinase domain active site, agreeing with the finding that EGCG competes for ATP binding, providing another important molecular mechanism for the anti-cancer activities of EGCG [67].

Protein phosphatase-1 (PP1) and protein phosphatase-2A (PP2A) are responsible for the dephosphorylation of most phosphoserine and threonine residues in cells. Kiss et al. showed that EGCG and penta-*O*-galloyl-β-d-glucose (PGG) inhibit the activity of the PP1 recombinant δ-isoform of the PP1 catalytic subunit and the native PP1 catalytic subunit (PP1c) with IC50 values of 0.47–1.35 μM and 0.26–0.4 μM, respectively. EGCG and PGG were weaker inhibitors of PP2Ac, with IC50 values of 15 and 6.6 μM, respectively. The finding suggests that the galloyl group may play a major role in the phosphatase inhibition. These studies using various techniques, including NMR, SPR, and MDA, found that EGCG docks at the hydrophobic groove close to the catalytic site of PP1c. This hydrophobic interaction is further stabilized by hydrogen bonding via hydroxyl/oxo groups of EGCG to PP1c residues, and EGCG binds to PP2A catalytic subunit in a similar manner, but in a distinct pose. EGCG suppressed the viability of HeLa cells, suggesting the EGCG binding to protein phosphatases may contribute to the anti-cancer effect of green tea [68].

Vascular endothelial growth factor (VEGF) is a factor involved in angiogenesis and is related to disease complications such as increased growth of tumors and atherosclerotic plaques. Moyle et al. showed that EGCG and apple procyanidin oligomers dp4 at nM concentrations inhibited VEGF-induced VEGF receptor-2 (VEGFR2) signaling in human umbilical vein endothelial cells by directly binding with VEGF. The VEGF activity could not be recovered by dialyzing the VEGF–polyphenol complexes. MDA predicted that EGCG and dp4 bind VEGF close to or in a region associated with receptor binding where they can interact with residues implicated in VEGFR2 binding. EGCG is located in a cavity between the two monomers of VEGF by the VEGFR2-binding site formed by Asp63 and Glu64 on one side, Tyr36, Ile43 and Ile46 on the other side with Ser30 and Asp34 forming the bottom of the cavity. Ile46 and Glu64 are two of the three residues that contribute mostly to the binding energy of VEGF to VEGFR2. dp4 binds to a region next to the cavity which EGCG occupies, to which dp4 is unable to have access presumably due to steric hindrance. dp4 also interacts with Glu64 with which dp4 can form a hydrogen bond. Additional residues identified as being part of the receptor binding face include Phe36, Lys48, Asn62 and Asp63, which may interact with EGCG or dp4. These findings may be useful in the designing anti-angiogenic drugs for cancer prevention [69].

Signal transducer and activator of transcription 3 (STAT3) is an oncogene that promotes cell survival, proliferation, and motility. Wang et al. studied the mechanism involved in the EGCG inhibition of STAT3 signaling using SPR-binding assays and MDA. They found that EGCG had a strong interaction with Arg609, one of the key residues contributing to STAT3 and phosphorylated peptide binding. EGCG inhibited cell proliferation of the hepatocellular carcinoma BE-7402 and QGY-7703 cells, induced apoptosis, lowered the expression levels of phosphorylated STAT3 proteins, and inhibited the expression of multiple genes, including BclxL, c-Myc, VEGF, and cyclin D1. These findings indicate that the anti-cancer function of green tea results, at least partly, from the EGCG’s inhibition of the STAT3 signaling [70].

### 4.2. Interaction between Catechins and MetS-Related Proteins

EGCG inhibits lipase activity, implicating its role in green tea’s prevention of certain diseases, including obesity and MetS. Wu et al. examined the interaction of EGCG and porcine lipase using various methods [71]. They found that EGCG bound to lipase with an affinity of Ka = 2.70 × 10^4^ L/mol. Circular dichroism studies indicated a conformational change of lipase upon binding to EGCG. MDA showed the hydrogen bonding interaction with Val21, Glu188, and Glu220. The noncovalent binding between EGCG and lipase is considered to alter the molecular conformation of lipase, leading to the decrease in its catalytic activity.

Similarly, Wang et al. observed that GTCs inhibited the activity of pancreatic lipase and that their inhibitory rates decreased in the order of EGCG > GCG > ECG > EC. α-Helix content of lipase secondary structure decreased concentration-dependently in the same order. Enzyme kinetic analyses showed that GTCs inhibited noncompetitively lipase activity without binding to the catalytic site. Differential scanning calorimetry showed that GTCs decreased the transition midpoint temperature of lipase, suggesting that these compounds reduced lipase thermostability [72].

HMGR is the rate-controlling enzyme of cholesterol biosynthesis, and many studies have been conducted to identify potential compounds capable of modulating its activity for developing hypocholesterolemic drugs. Utilizing a concerted approach with various methods, including MDA and site-directed mutagenesis on the cofactor NADPH site of HMGR, Cuccioloni et al. demonstrated that EGCG potently inhibited the enzymatic activity with Kd values of <100 nM by competitive binding at the NADPH site. The EGCG-HMGR complex was characterized by the putative formation of 11 hydrogen bonds involving 10 amino acid residues, Glu559, Asp690, Lys691, Gln770, Val805, Gly807, Met659, Met655, Asn658, and Asp767. The hydrogen bond formation involving Met659, Met655, Asn658, and Asp767 was a common feature with NADPH. The G807D mutant retained the HMGR enzymatic activity, indicating that Gly807 does not influence catalytic activity, but showed the nearly abolished capacity of the mutant to bind EGCG [73].

Islam et al. showed that GTCs bind to HMGR and block the binding of NADP^+^. The study also showed that the rigidity of phenolic rings prevents the polyphenols from docking to the enzyme activity site and that an ester linkage between the phenolic rings in EGCG allows EGCG to orientate in the active site and bind to the catalytic residues of HMGR. The finding suggests that direct binding of EGCG to HMGR may be involved in the hypolipidemic effect of green tea, which has been reported in several epidemiological and animal studies [74].

The microsomal enzyme 11β-hydroxysteroid dehydrogenase type 1 (11βHD1) acts as an intracellular switch for regulating the access of glucocorticoid hormones to the glucocorticoid receptor and is associated with insulin resistance, type 2 diabetes, hypertension, dyslipidemia, and obesity. Inhibitors of 11βHD1 may be a target of a therapeutic approach for the MetS. Hintzpeter et al. found that EGCG inhibited 11βHD1-mediated cortisone reduction. Kinetic studies indicated a direct competition mode of EGCG, with the substrate and cofactor binding. Inhibition constants of EGCG on cortisone reduction were Ki = 22.68 μM for microsomes and Ki = 18.74 μM for purified 11βHD1. MDA showed that EGCG binds directly to the active site of 11βHD1 by forming a hydrogen bond with Lys187 of the catalytic triad. EGCG’s inhibition of 11βHD1 may contribute to the beneficial effect of green tea on MetS [75].

In search of novel angiotensin converting enzyme (ACE) inhibitors, Ke et al. utilized MDA and a rigorously validated model based on a natural compounds database. They selected 36 compounds randomly and subjected them to an in vitro ACE kinase inhibitory assay using a fluorescence method. The results showed that three compounds, including EGCG, have a strong potential to be developed as a new class of ACE inhibitors. Further chemical modification via fragment modifications guided by the structure and ligand-based computational methodologies would lead to better agents being discovered as potential clinical candidates [76].

Menegazzi et al. demonstrated that EGCG and ECG bound to the signal transduction activator of transcription 1 (STAT1) with Kd = 23 nM in breast cancer cells [77]. Previous studies showed that EGCG inhibited STAT1 activation. The data obtained by techniques, including SPR and MDA, indicated that the presence of three hydroxyl groups of the B ring and one hydroxyl group on the D ring of EGCG are essential to exert the inhibitory action and that there are two putative binding sites (A and B) with different affinities. The data also indicated that His568 on site B could be important in the catechin–STAT1 interaction because this site showed higher affinity than site A, and the EGCG’s binding to STAT1 was markedly diminished in the mutant in which His568 was replaced with Ala568 [73]. These findings provide a molecular basis for the EGCG’s health benefit such as green tea’s cardioprotection through inhibition of STAT-1 activity. A similar molecular basis may be applied in the clinical setting to minimize STAT-1 activation levels in patients with acute coronary artery disease [78].

### 4.3. Interaction between Catechins and Inflammation-Related Proteins

TGFβ-activated kinase 1 (TAK1) plays a key role in interleukin (IL)1β, TNF, and Toll-like receptor signaling. Singh et al. found that inhibition of TAK1 completely abrogated IL1β-induced IL6 and IL8 syntheses in human rheumatoid arthritis synovial fibroblasts. Experiments using methods, including MDA, showed that EGCG inhibited TAK1 phosphorylation at Thr184 and Thr187 and occupied the Cys174 position, an ATP binding site, to inhibit the kinase activity. EGCG pretreatment also inhibited Lys63-linked autoubiquitination of TRAF6 by forming a stable hydrogen bond at the Lys124 position on TRAF6. Western blot analyses of joint homogenates from rats with adjuvant-induced arthritis showed a significant increase in Lys48-linked polyubiquitination, TAK1 phosphorylation, and TRAF6 expression as compared with naive rats. Administration of EGCG (50 mg/kg/day) for 10 days ameliorated adjuvant-induced arthritis by reducing TAK1 phosphorylation and Lys48-linked polyubiquitination. These findings imply that EGCG may be an agent for targeting TAK1 in the treatment of rheumatoid arthritis [79].

Fechtner et al. found that EGCG, EGC, and EC differentially interfere with the IL1β signaling pathway which regulates the expression of pro-inflammatory mediators (IL6 and IL8) and COX2 in primary human rheumatoid arthritis synovial fibroblasts. EGCG and EGC inhibited IL6, IL8, and MMP2 production and selectively inhibited COX2 expression. EC did not exhibit any inhibitory effects. These catechins inhibited TAK1 activity to contribute to the anti-inflammatory effect of green tea. MDA confirmed that although EGCG, EGC, and EC all occupy the active site of the TAK1 kinase domain, EGCG occupies most the TAK1 active site. EGCG also inhibited P38 and NFκB expression, whereas EC and EGC were not effective inhibitors. These findings suggest that EGCG is the most effective catechin in inhibiting downstream inflammatory signaling, but its effectiveness could be hindered by the presence of EC. Therefore, EC content in green tea may affect the anti-inflammatory efficacy of green tea [80].

### 4.4. Interaction between Catechins and Microbial Proteins

Previously, Toda et al. found that tea catechins inhibited the activity of a cholera toxin (CT), and protect against cholera infection in an animal model [81]. CT is a toxin which is composed of a catalytic A1 subunit, an A2 linker, and a cell-binding B subunit. Cherubin et al. found that although EGCG inhibited the toxic activity of CT, binding was not affected between the B subunit pentamer and plasma membrane of Vero cells pre-treated with EGCG. The finding suggests that EGCG binds to the toxin rather than the host cell surface. MDA identified potential EGCG binding sites in CT as the GM1 ganglioside-binding pocket of B subunit [82].

Envelope glycoprotein E (EGE) of Dengue virus (DENV) mediates viral attachment and entry into the host cells. Vazquez-Calvo et al. identified EGCG’s virucidal effect against DENV. MDA predicted the binding of nine flavonoids (EGCG, baicalin, baicalein, fisetin, glabranine, hyperoside, ladanein, quercetin, and flavone) to the soluble ectodomain of DENV type 2 (DENV2) EGE. Eight flavonoids, including EGCG, were found to dock into the same binding pocket located between the domain I and domain II of different subunits of EGE. Similar docking results were observed for the EGE structures of other virus types. MDA showed Ile4, Gly5, Asp98, Gly100 and Val151 residues of the DENV2-Malaysia EGE that aligned with the same residues in the DENV2-Thai EGE can form consistent hydrogen bond interactions with EGCG, baicalein, and quercetin during the simulations. This study shows interactions of these flavonoids with the EGE of DENV2, leading to the inhibition of viral infection [83].

Human papillomavirus (HPV) infection is the leading cause of cancer mortality among women worldwide. EGCG has been shown to suppress HPV infection. Kumar et al. explored natural inhibitors against E6 oncoprotein of high-risk HPV16, which is known to degrade the p53 tumor suppressor protein. MDA indicated the interaction of EGCG with the p53-binding site of E6 protein residues 113–122 (Cys-Gln-Lys-Pro-Leu-Cys-Pro-Glu-Glu-Lys) and helped the restoration of p53 functioning. EGCG was found to interact with E6 oncoprotein with binding energies of −4.13 kcal/mol. Leu106, Ile108, and Leu117 were identified as the interacting residues of the receptor proteins with EGCG [84].

### 4.5. Interaction between Catechins and Proteins Related to Neurodegenerative Diseases

Alzheimer’s disease (AD) is a progressive neurodegenerative disorder, which destroys the intellectual and cognitive abilities. HSP90α is involved in regulating the function of tau proteins, which are involved in the cause of the AD. Inhibition of HSP90α by C-terminal domain ATP binding site blockage may be an effective treatment strategy against the disease via degradation of tau proteins. Khalid and Paul performed MDA and showed that Leu665, Leu666, and Leu694 could be the binding sites of HSP90α and the HSP90 inhibitors such as EGCG. The best binding energy was lower at Leu666 (−7.53 kcal/mol) than those at Leu665 (−7.20 kcal/mol) and Leu694 (−6.67 kcal/mol). Based on a recent report which shows that EGCG enhances the clearance of phosphorylated tau protein in primary neurons, EGCG may be considered to be a promising therapeutic agent in AD [85].

The accumulation of β-sheet-rich amyloid fibrils or aggregates is associated with cellular toxicity in several human protein misfolding disorders, including AD and Parkinson’s disease. Ehrnhoefer et al. demonstrated redirection of amyloid fibril formation through the action of a small molecule. EGCG efficiently inhibits the fibrillogenesis of both α-synuclein and amyloid β (Aβ) by directly binding to the natively unfolded polypeptides and preventing their conversion into toxic aggregation intermediates. At equimolar concentrations, EGCG preferentially bound the C-terminus of α-synuclein (Asp119, Ser129, Glu130, and Asp135). EGCG promoted the formation of unstructured, nontoxic α-synuclein and Aβ oligomers of a new type, instead of β-sheet-rich amyloid, suggesting its favorable effect on aggregation pathways in neurodegenerative diseases [86].

The Aβ oligomers have been shown to be the main culprits in the cytotoxicity of the AD, and p3 peptides (Aβ17-42 fragments) are present in AD amyloid plaques. Chebaro et al. have determined the structure of Aβ17-42 trimers both in aqueous solution and in the presence of five small molecule inhibitors including EGCG, 2002-H20, curcumin, naphthoquino-2-yl-tryptophan, and resveratrol. Replica exchange molecular dynamics simulations coupled to the protein coarse-grained optimized potential for efficient structure prediction found that the conformational ensemble of Aβ17-42 trimer can be described by 14 clusters with each peptide essentially adopting turn/random coil configurations. However, the most populated cluster is characterized by one peptide with a β-hairpin at Phe19–Leu31. The dock simulations revealed that the drugs have multiple binding modes with different binding affinities for trimeric Aβ17-42, although they interact preferentially with the region of amino acid residues 17–21 [87].

The pathogenic aggregation of the Aβ peptide is one of the hallmarks of the progression of AD leading to senile dementia. In their comprehensive review article, Lemkle and Bevan have discussed how technologies such as MDA and molecular dynamics simulations can be applied in developing small molecules as therapeutics for the prevention of Aβ. These techniques showed that EGCG binds to Aβ42, revealing 12 amino acid residues to which EGCG bound primarily. Polar interactions such as hydrogen bonding play only a minor role within this process, and a 10-fold excess concentration of EGCG was capable of preventing the increase of a β-strand content in the polypeptide, leading to the inhibition of aggregation. Exclusion of water from the surface of Aβ and the resulting interactions between EGCG and Aβ may be responsible for the inhibition of structural change. Thus, the affinity of EGCG for many residues in the Aβ sequence could explain its strong inhibitory effect toward aggregation [88].

Hyung et al. compared the interactions and reactivities of EGCG with metal (Cu(II) and Zn(II))-Aβ (mAβ) and metal-free Aβ peptides and found that EGCG interacted with mAβ and formed small, unstructured Aβ aggregates more distinctively than in metal-free conditions. EGCG attenuated the toxicity of both metal-free Aβ and mAβ in cultured neuroblastoma cells. Experiments using ion mobility-mass spectrometry, 2-dimentional NMR spectroscopy, and MDA found that EGCG was bound to Aβ monomers and dimers to form more compact peptide conformations than those from EGCG-untreated Aβ species and that ternary EGCG-Aβ complexes were produced. These findings demonstrate the anti-amyloidogenic activity of EGCG toward mAβ species with a structure-based mechanism [89].

## 5. X-ray Crystallographic Analysis of the Catechin–Protein Complex

### 5.1. Interaction between Catechins and Cancer-Related Proteins

**Figure 4:**
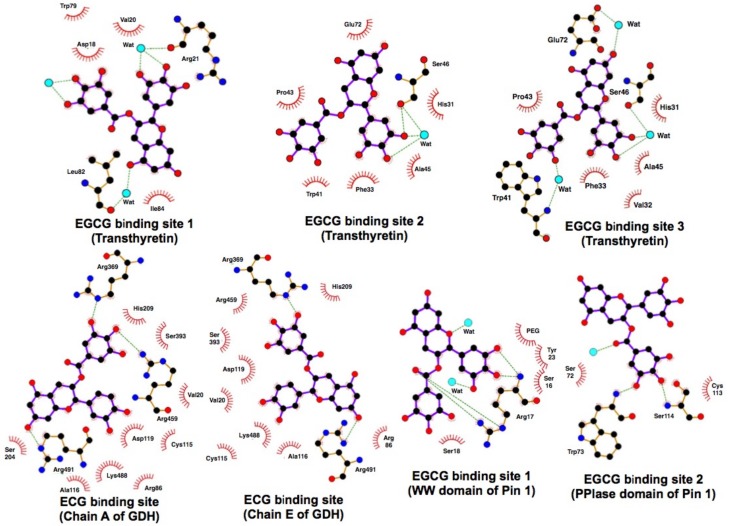
X-ray crystal structure analysis of protein/GTC complexes [28,85,86]. The protein/ligand contacts are schematized using the protein data bank (PDB) (code: 3NG5, Transthyretin; 3QMU, GDH; 3OOB, Pin1) and LIGPLOT+ program [92]. Hydrogen bonds are displayed as dotted green lines and van der Waals interactions as red stellations. The water molecules are shown as cyan spheres.

As described above, pull-down with EGCG-agarose showed the binding interaction between EGCG and Pin1 which plays a critical role in oncogenic signaling. X-ray crystallography confirmed the direct binding of EGCG to Pin1 and the presence of EGCG in two conserved tryptophan domains: (1–39) and peptidyl-prolyl isomerase domain (45–168) [30] (Figure 4). This interaction resulted in the inhibition of Pin1 isomerase activity. EGCG inhibited the proliferation of cells expressing Pin1 and suppressed tumor growth in a xenograft mouse model. The binding of EGCG with Arg17 in the domain (1–39) prevented the binding of substrate c-Jun. EGCG caused a decreased abundance of cyclin D1 and decreased activator protein 1 or NFκB promoter activity induced by a phorbol ester in cells expressing Pin1. These results show that EGCG directly suppresses the tumor-promoting effect of Pin1.

Glutamate dehydrogenase (GDH) catalyzes the oxidative deamination of L-glutamate and its mutations that abrogate GTP inhibition; this leads to the hyperinsulinism/hyperammonemia syndrome (HHS), resulting in increased pancreatic β-cell responsiveness to leucine and susceptibility to hypoglycemia following high-protein diets. Li et al. showed that EGCG and ECG inhibited GDH, and that EGCG blocked GDH-mediated insulin secretion in wild-type rat islets [90]. In their structural and site-directed mutagenesis studies, ECG was shown to bind to the same site as the allosteric regulator, ADP (Figure 4). Using pancreatic islets from transgenic mice expressing a human HHS form type, EGCG was demonstrated to block the hyper-response to glutamine caused by dysregulated GDH. Prior oral administration of EGCG abrogated hypersensitivity to amino acid feeding. Chronic administration of EGCG improved the low basal blood glucose concentration in this mouse model. These findings suggest that restriction of glutamine catabolism via GDH inhibitors such as EGCG can be useful in treating various tumors.

### 5.2. Interaction between Catechins and Aamyloidosis Protein

As already mentioned, in neurodegenerative diseases such as AD and Parkinson’s disease, aberrant amyloid fibril formation is associated with protein misfolding. Familial amyloid polyneuropathy is a disease caused by a point mutation of transthyretin (TTR), a transporter of thyroxine. Amyloid fibrils derived from TTR variants contribute to the pathogenesis of amyloidosis. EGCG has been shown to bind to TTR and suppress the amyloid fibril formation. Miyata et al. investigated the crystal structure of the complex of EGCG-TTR mutant with V30M and found novel binding sites distinct from the thyroxine binding site [91] (Figure 4). EGCG induced the oligomerization and monomer suppression in the clinical variants of TTR. These findings suggest that EGCG may be a candidate therapeutic compound for familial amyloid polyneuropathy.

## 6. Conclusions

EGCG has been shown to contribute to a variety of beneficial health effects of green tea. EGCG may act as an anti-oxidant and paradoxically as a pro-oxidant. Meanwhile, many studies have found that the EGCG-protein binding interaction is involved in such beneficial health effects. In the early stage of these investigations, AGC and pull-down experiments using EGCG-agarose beads played important roles. MDA has confirmed several of these experimental results. SPR contributed to the discovery of cell surface EGCG receptor 67LR, which opened a new avenue to the understanding of EGCG’s actions. MDA and X-ray crystallographic analyses have yielded a great deal of information on the catechin–protein binding interaction. This information may be useful in rational drug design of EGCG derivatives for various protein targets. These derivatives may become new drugs for clinical trials.

The studies described here on EGCG-protein binding interaction have demonstrated how EGCG can fit into the active site, or bind close to an active site and induce a conformational change. The blocking of the active site, steric hindrance by EGCG bound near an active site, or conformational change caused by the EGCG binding, would lead to the inhibition of enzymatic activity and other biological activities of proteins. Such binding may be related to EGCG’s favorable effects including those in cancer, MetS, and inflammation. The weak effects of EC and EGC can be explained by the absence of a gallate residue which plays an important role in this binding. In addition, binding of EGCG to fibrous proteins can inhibit the formation of their toxic aggregates and prevent neurodegenerative diseases and amyloidosis. Future studies will provide further evidence to give a rationale for EGCG therapy and for EGCG to be a lead compound for the rational design of new drugs.

## Figures and Tables

**Table 1:** Binding Interaction between EGCG and protein as revealed by affinity gel chromatography (AGC) and/or pull-down (PD) methods with EGCG-conjugated agarose.

Experimental Mode	Protein	Binding Characteristics	Head Author (Year)	Reference
AGC	Fibronectin	EGCG binds to the carboxyl-terminal heparin-binding domain.	Sazuka, M. (1996;1998)	[21] [22]
AGC	MMP-2 *	EGCG binding to MMP-2 was identified by gelatin zymography.	Sazuka, M. (1997)	[32]
AGC	MMP-9 **	EGCG binding to MMP-9 was identified by gelatin zymography.	Sazuka, M. (1997)	[32]
AGC, PD	Vimentin	EGCG binds to the region of 50–63 residues.	Ermakova, S. (2005)	[33]
PD	HSP90 **	EGCG binds to a C-terminal geldanamycin binding site (amino acid residues 538–728)	Palermo, C.M. (2005) Moses, M.A. (2015)	[23] [24]
PD	GRP78 **	EGCG binds to the ATPase catalytic domain (211–654 residues)	Ermakova, S.P. (2006)	[25]
PD	IGF1R **	The participating residues in the binding include Gln977, Lys1003, MEet1052, The1053, and Asp1123EGCG binds to the ATP binding pocket in β-subunit.	Li, M. (2007)	[26]
PD	Fyn	EGCG binds to the SH2 domain, but not the SH3 domain	He, Z. (2008)	[27]
PD	ZAP70 **	EGCG binds to an ATP binding siteGlu415, Ala417, Lys369, Asp479, Glu386.	Shim, J.H. (2008)	[28]
PD	G3BP1	EGCG binds to the region of amino acid residues 226–340.	Shim, J.H. (2010)	[29]
PD	Pin1 ***	EGCG bound to WW domain with two conserved tryptophans (1–39) pSer/Thr–Pro recognition loop of Met15–S16-R17-S18-R21-Tyr23 and to the peptidyl prolyl isomerase domain of Pin. EGCG creates several strong contacts with Pin1 at Asp112, Ser114, Trp73, and Ser114.	Urusova, D.V. (2011)	[30]
PD	TRAF6 **	EGCG binds to TRAF6 at the residues of Gln54, Gly55, Asp57 ILe72, Cys73 and Lys96. Mutation of Gln54, Asp57, ILe72 in TRAF6 destroys EGCG binding to TRAF6.	Zhang, J. (2016)	[31]

* The binding interaction with EGCG as revealed by MDA is depicted in Figure 3; ** The binding interaction with EGCG was confirmed by MDA (Section 4); *** The binding interaction with EGCG as revealed by X-ray crystallography is depicted in Figure 4.

## Abbreviations

11bHD1	11β-hydroxysteroid dehydrogenase type 1
67LR	67-kDa laminin receptor
Aβ	amyloid β protein
ACE	angiotensin converting enzyme
AD	Alzheimer’s disease
AGC	affinity gel chromatography
AhR	aryl hydrocarbon receptor
AMPK	AMP-activated protein kinase
aSMase	acid sphingomyelinase
Bcl2	B-cell lymphoma-2
BclxL	B-cell lymphoma-extra large
BSA	bovine serum albumin
CT	cholera toxin
CXCL	C-X-C motif chemokine ligand
DENV	Dengue virus
DHFR	dihydrofolate reductase
DNMT	DNA methyltranseferase
EC	(−)-epicatechin
ECG	(−)-epicatechin gallate
EGC	(−)-epigallocatechin
EGCG	(−)-epigallocatechin-3-*O*-gallate
EGE	envelope glycoprotein E
EGF	epidermal growth factor
FASN	fatty acid synthase
G3BP1	SH3 domain-binding protein 1
GAP	GTPase-activating protein
GBM	glioblastoma
GDH	glutamate dehydrogenase
GLIDE	grid-based ligand docking with energetics
GRP78	glucose-regulated protein 78 kDa
GST	glutathione-*S*-transferase
GTCs	green tea catechins
HDAC	histone deacetylase
HFD	high fat diet
HHS	hyperinsulinism/hyperammonemia syndrome
HMGR	hydroxymethyl-glutaryl-CoA reductase
hMLH1	human mutL homologue 1
HPV	human papillomavirus
HSP	heat shock protein
IGF1R	insulin-like growth factor 1 receptor
IL	interleukin
MAPK	mitogen-activated protein kinase
MDA	molecular docking analysis
MetS	metabolic syndrome
MMP	matrix metalloproteinase
MT1-MMP	membrane-type 1 matrix metalloproteinase
NF-κB	nuclear factor-kappa B
PGG	penta-*O*-galloyl-β-d-glucose
PI3K	phosphoinositide-3-kinase
Pin1	peptidyl prolyl cis/trans isomerase 1
PP1	protein phosphatase-1
PP2A	protein phosphatase-2A
QCM	quartz crystal microbalance
RARβ	retinoic acid receptor beta
ROS	reactive oxygen species
SH3	Src homology 3
SPR	surface plasmon resonance
SREBP	sterol-response element binding protein
STAT	signal transducer and activator of transcription
TAK1	transforming growth factor β-activated kinase 1
TGFRII	transforming growth factor β type II receptor
TGFβ	transforming growth factor β
TNF	tumor necrosis factor
TRAF6	TNF receptor associated factor 6
TTR	transthyretin
Ubc13	ubiquitin-conjugating protein 13
VEGF	vascular endothelial growth factor
VEGF1R	vascular endothelial growth factor 1 receptor
VEGFR2	VEGF-induced VEGF receptor-2
ZAP-70	ζ chain-associated 70 kDa protein
